# Exploring Entertainment Medicine and Professionalization of Self-Care: Interview Study Among Doctors on the Potential Effects of Digital Self-Tracking

**DOI:** 10.2196/jmir.8040

**Published:** 2018-01-12

**Authors:** Katleen Gabriels, Tania Moerenhout

**Affiliations:** ^1^ Philosophy & Ethics, Department of Industrial Engineering & Innovation Sciences Eindhoven University of Technology Eindhoven Netherlands; ^2^ Ethics, Autonomy and Responsibility in Health Care Department of Family Medicine and Primary Health Care Ghent University Ghent Belgium; ^3^ Department of Philosophy and Moral Sciences Ghent University Ghent Belgium

**Keywords:** mobile applications, wearable electronic devices, self-recorded health data, self care, quantified self, qualitative research

## Abstract

**Background:**

Nowadays, digital self-tracking devices offer a plethora of possibilities to both healthy and chronically ill users who want to closely examine their body. This study suggests that self-tracking in a private setting will lead to shifting understandings in professional care. To provide more insight into these shifts, this paper seeks to lay bare the promises and challenges of self-tracking while staying close to the everyday professional experience of the physician.

**Objective:**

The aim of this study was to (1) offer an analysis of how medical doctors evaluate self-tracking methods in their practice and (2) explore the anticipated shifts that digital self-care will bring about in relation to our findings and those of other studies.

**Methods:**

A total of 12 in-depth semistructured interviews with general practitioners (GPs) and cardiologists were conducted in Flanders, Belgium, from November 2015 to November 2016. Thematic analysis was applied to examine the transcripts in an iterative process.

**Results:**

Four major themes arose in our body of data: (1) the patient as health manager, (2) health obsession and medicalization, (3) information management, and (4) shifting roles of the doctors and impact on the health care organization. Our research findings show a nuanced understanding of the potentials and pitfalls of different forms of self-tracking. The necessity of contextualization of self-tracking data and a professionalization of self-care through digital devices come to the fore as important overarching concepts.

**Conclusions:**

This interview study with Belgian doctors examines the potentials and challenges of self-monitoring while focusing on the everyday professional experience of the physician. The dialogue between our dataset and the existing literature affords a fine-grained image of digital self-care and its current meaning in a medical-professional landscape.

## Introduction

Today, individuals are offered a multitude of possibilities to track and so manage their personal health. Wearables such as Jawbone and Fitbit allow people to monitor bodily processes and activities. Self-tracking through wearable and otherwise mobile computing has become known as the *quantified self* [1]. Quantified self technologies—usually small computers that record data—provide the individual with detailed information including sleeping habits and calories burnt. This knowledge can lead to changes in self-understanding [2].

Although self-care has been an established practice for several years (eg, the use of home blood-pressure monitors), digital self-care by means of mobile computing—so-called mobile health (mHealth)—is still in its introductory stages. As an initial step to understanding the opportunities, as well as ethical challenges that mHealth tools present, we examine how both classic and digital self-tracking methods are incorporated into daily health care.

Classic self-tracking methods provide support to enhance self-care: “the care of oneself without medical, professional, or other assistance or oversight” [3]. The concept is complex, encompassing various aspects, and changing over time. Here is a more comprehensive definition [3]:

Self-care is deliberate care performed throughout life; by individuals to themselves and to others; to promote health or improve both general health and mental health, and cope with illness or disability; and in collaboration with healthcare professionals or performed separately. Self-care also includes social support and provides the continuity of care necessary to maintain wellbeing.

When focusing on the management of chronic disease, the term “self-management” is appropriate, defined as the “patient’s ability to manage the symptoms, treatment, physical and psychosocial consequences, and lifestyle changes inherent in living with a chronic condition” [4]. The application of self-analytic methods is not new. Indeed, physicians have been recommending self-tracking for some time, both for self-management (eg, diabetes) and professional care (eg, cardiac monitoring). Before apps and wearable technologies became widespread, self-tracking for self-care relied on more simple technologies such as thermometers and bathroom scales.

Researchers investigating the application of digital self-tracking to professional care vary from the enthusiastic, focusing on the technology’s revolutionary promise [5], to the skeptical, focusing on the risks [6]; with any number taking moderate standpoints in between [7]. Health apps and devices promise to discover medical problems earlier and also serve to coach the user toward behavioral changes: for example, more exercise. Self-tracking technologies are appropriate both for healthy persons (preventive care) and for the ill (interventionist or therapeutic use)—in which case, the technology assists in keeping a close watch on the condition, noting any anomalies. In both cases, the data can be shared with a professional health care provider.

This paper has a twofold objective: first, to analyze how medical doctors evaluate both classic and digital self-tracking on an everyday clinical basis. Toward this end, we conducted semistructured interviews with Belgian general practitioners (GPs; n=7) and cardiologists (n=5) to gain insight into their shifting understandings of these technologies. How do they incorporate self-tracking in their daily practice? What ethical issues do they encounter? What are their expectations for the future of digital self-tracking? Second, we sought to explore the transitions that digital self-tracking will bring about in self-care and professional health care by relating existing literature to care providers’ everyday experience.

Throughout this paper, classic is distinguished from digital self-tracking based on two main characteristics: data sharing and who takes the initiative to track. First, in the case of classic self-tracking, data are usually collected for private use only, whereas sharing is generally limited to care providers and mostly paper-based. The care provider typically initiates the request to self-track and, at least in some cases, provides the device or gives recommendations on which device to use and how to track (*cf*. “pushed” self-tracking, ie, the incentive to self-track comes from an external actor [8]). Second, digital self-tracking is most often Internet-connected, mobile phone–based, and designed from the start for data sharing. Members of the quantified self movement share their data with each other through social media (*cf.* “communal” self-tracking, ie, the practice of sharing data with a community of trackers [8]). It is usually the patient or healthy user who initiates the tracking. In some cases, the app takes over the care provider’s role. Coaching apps are designed to motivate behavioral change. Collected data can easily be shared with care providers and become part of patients’ electronic health record (EHR). The distinction of classical from digital self-tracking is not clear-cut. Rather, it is situated on a continuum, which facilitates comparison.

## Methods

### Sample Selection

This study used purposive, convenience-based sampling to select care providers, aiming at maximum variation in expertise, gender, years of experience, geographical location, and type of practice. We selected doctors with relevant experience with classic self-tracking. The heterogeneous sample served to develop, first, a broader insight into classic and digital self-tracking in everyday health care and, second, a range of perspectives by viewing the matter through various lenses. One author (TM), who works part-time as a GP, facilitated the recruiting process. This helped us gain access to this specific group of experts who are often hard to reach. In qualitative studies such as ethnographies, it is not uncommon to conduct research in the group one already has access to or in the group one already is a part of [9].

We arrived at a point of saturation after seven interviews [10,11]. Our participants shared similar views on most of the topics we talked about throughout the interviews and raised similar themes, suggesting that we reached a point where we could reasonably expect to not collect any further contrasting results, even within our small sample. Despite their experience with classic self-tracking, the GPs had little experience with digital self-tracking, and this was not often brought up by patients. Digital self-care is not yet an integrated part of their practice.

We subsequently interviewed cardiologists selected on the basis of experience with digital self-tracking and telemonitoring (in their case, remote tracking of patients with an implanted pacemaker or defibrillator). We arrived at a point of saturation after five interviews.

The Ethical Commission of Human Sciences (Vrije Universiteit Brussel) approved the study. All respondents are Belgian and Dutch-speaking. They gave their consent to participate and were informed about the researchers and research context. The respondents signed an informed consent and consented to a digital audio recording (see Multimedia Appendix 1 for the informed consent form). All interviews were conducted face-to-face and took place in the office of the physicians.

### Interview Guide

The interviews were semistructured: the interviewer started from an interview guide with a set of predetermined topics but equally left scope for extra questions or topics (see Multimedia Appendix 2). The interview guide for GPs was divided into two sections. We started with questions aimed at understanding the current situation: how do the providers incorporate classic self-tracking methods in their everyday practice? We focused on blood pressure meters, oximeters, and glucose meters as concrete examples. The questions were organized into five topics of which the last was the most elaborately discussed topic:

1. Frequency of use
2. Initiative to self-track
3. Choice and quality of device
4. Data interpretation
5. Positive and negative results or effects of self-tracking

The second part of the interview focused on digital self-tracking. The GPs were presented with five examples of apps. The questions were organized in the same manner as the first part of the interview, with three additional topics:

1. Differences between classic and digital self-tracking
2. Data sharing
3. Inclusion (health disparities)

We ended the interview with a peek into the future, asking them to imagine their practice 10 years from now and the evolution they expected in self-tracking and the health care organization.

The interview guide developed for cardiologists was similar to the first one, except for minor changes that made it more relevant to the cardiology practice, for example, focusing on blood pressure and heart rate apps.

### Data Analysis

All interviews were transcribed ad verbatim by one of the researchers (KG transcribed 7 interviews and TM 5). We performed a thematic analysis carried out in three phases. The *first phase* involved labeling and tagging the data and assigning codes to the text by marking words and phrases in Word (Microsoft) [12]. The first interview was coded by both researchers, followed by a thorough discussion of the developed codes until consensus was reached. One researcher (TM) continued to code all interviews in an iterative process. The second researcher (KG) independently coded three more interviews to ensure validity and reliability of the codes. This was again followed by an elaborate discussion of code definition until consensus was reached. Both authors were also particularly attentive to deviant cases in the interviews. A coding scheme was subsequently developed that first derived *deductive* codes based on the literature review and research questions. Second, the coding scheme also consisted of *inductive* codes, emerging directly from the interviews (see Multimedia Appendix 3 for the coding scheme).

The *second phase* consisted of an in-depth search for deeper themes in the codes. In doing so, the data were recoded thematically, and patterns were looked for between the codes to identify and generate core and deeper themes [13].

The *third phase* involved the move from themes to theory: we linked the themes with academic literature on self-care and embedded them in broader frameworks. The selected quotes were translated from Dutch to English by the researchers. One researcher (KG) also has a master’s degree in English (linguistics and literature), which helped assure an accurate translation.

To enhance quality, reliability, and validity of the research, we also included member validation [14]. Three cardiologists and four GPs of our sample reviewed a version of this manuscript and its research findings. We invited them to comment upon our interpretations [15]. All of them agreed with our study findings. Some participants gave a number of constructive suggestions; we subsequently took their remarks into consideration and included them in the manuscript.

### Interviews

All interviews were conducted from November 2015 to November 2016. One researcher (TM) carried out eight interviews (six GPs and two cardiologists); the second researcher (KG) conducted four interviews (one GP and three cardiologists). The shortest interview was 38 min (transcript of 5400 words), and the longest was 105 min (transcript of 14,200 words). As described above, we only selected GPs who had extensive experience with classic self-tracking in their daily practice and aimed for a heterogeneous sample. One GP has specific experience as an information technology (IT) expert for a local GP network. Cardiologists 11 and 12 are also engaged in research projects on this subject. A schematic overview of the sample can be found in Table 1.

**Table 1:** Characteristics of the sample.

Number	Occupation	Gender	Age range (years)^a^	Years of experience	Type of practice
1	General practitioner	Female	26-30	2	Duo
2	General practitioner	Female	31-35	4	Group
3	General practitioner	Female	36-40	9	Solo
4	General practitioner	Male	51-55	7	Solo
5	General practitioner	Female	56-60	30	Solo for 26 years, now group
6	General practitioner	Female	41-45	15	Health community center
7	General practitioner	Male	66-70	40	Duo
8	Cardiologist	Female	46-50	15	Hospital
9	Cardiologist	Male	56-60	25	Hospital
10	Cardiologist	Male	36-40	4	Hospital
11	Cardiologist	Female	26-30	2 (assistant)	Hospital and research
12	Cardiologist	Male	56-60	20+	Hospital and research

^a^ For the sake of confidentiality, we opted for providing the age range instead of the year of birth.

### Belgian Situation

Given that the interviews are conducted with Belgian health care providers, we concisely describe the Belgian health care context. Belgium is an active welfare state with an extensive form of social security, covered by social contributions based on income [16]. One of the six sectors of the social security system is a compulsory health insurance with a broad benefits package covering almost the entire population [17]. Costs are either paid by the patient who is reimbursed afterwards (direct payment system, often the case for primary care) or paid directly by the government, except for the copay or nonrefundable part (third-party payer system, mostly for secondary and tertiary hospital care) [17]. Generally speaking, insurance is provided in a hybrid single-payer (with broad coverage) and private insurance system (for additional coverage). Notwithstanding minor differences, the Belgian health care system can be compared with that of France, Germany, the United Kingdom (and most other European countries), and Australia. Although the United States may be different in terms of health insurance, with a stronger emphasis on the private insurance system, it also faces the same questions in terms of apps and wearables.

Currently, there is no fee paid to providers for coaching or follow-up of self-tracking, only for telemonitoring of implanted defibrillators and pacemakers. In 2016, Maggie De Block, the Belgian minister of social affairs and health, announced that she would provide funding for a number of pilot projects in the context of mobile health to investigate the reimbursement for use of specific health apps and devices [18]. In so doing, a matrix needs to be developed for criteria and qualifications, as well as a CE (European conformity) label. For instance, instead of prescribing a sleeping pill, a doctor might prescribe use of a sleep-monitoring app, with the costs subsequently reimbursed by the health insurance.

## Results

### Overview

Four major themes arise in our body of data:

The patient as health manager: patients are offered numerous possibilities to control and self-manage their health, leading to both opportunities and difficulties. Subthemes are patient autonomy, dropout rates, and the gap between measuring and attaining actual behavioral changes.

Health obsession: the interviewees express concern about a focused use of self-tracking by healthy people, thus creating a “worried well” cohort and widening health disparities. They are critical of the broader medicalization trend in society. Another subtheme is “entertainment medicine,” which refers to questioning the usefulness of digital self-tracking in terms of medical necessity.

Information management: data production, analysis, and interpretation methods change with intensified self-tracking. In this context, providers describe opportunities but also new pitfalls. Subthemes are quality and reliability (of devices and data), importance of context, and data sharing.

Shifts in the roles of the doctor and impact on the health care organization: the impact of digital self-care data on the clinical practice, leading to shifts in terms of data interpretation and the role of the physician. Subthemes are data overload, responsibility, and the importance of in-person contact.

The results show a nuanced and multifaceted understanding of the promises and drawbacks of self-tracking. Findings reveal that digital self-tracking is still emerging and not yet a standard part of the clinical practice, even though the technology is readily available. The interviewed GPs do not often encounter (questions about) new self-tracking technology. In the cardiologist practice, home monitoring (telemonitoring) of defibrillators and pacemakers is already well-established; the cardiologists have firsthand experience with data analysis and complexity of digital health care, although self-care with apps and wearables is not yet an integrated part of their practice and consultation. Just like the GPs in our sample, the cardiologists are not often confronted with patient-initiated forms of self-tracking (eg, digital heart rate monitoring).

### Overview of Themes

#### Theme 1: The Patient as Health Manager

A nuanced sketch of the patient-manager, emerging from the possibilities of self-tracking, is a key theme throughout all the interviews. The digitalization of self-care can be an empowering tool for patients to actively manage their health. Informants agree that classic forms of self-tracking induce a feeling of control in patients that may lead to an increased quality of care.

Some people also like to have control over their health and it is not a bad thing that there are methods to meet this demand. Many people are perfectly capable of doing this. You do not need to have studied for nine years to know what high blood pressure or glucose means, and what you can do about it.

2, GP

Self-tracking tools allow patients to monitor and adjust their lifestyle personally, which gives them more self-determination and autonomy with regard to their health. Providers acknowledge that self-tracking technologies can offer more insight into bodily information, which opens interesting paths for preventive screening and lifestyle interventions. Patients with unhealthy habits such as smoking can use the technologies as a coach toward a healthier lifestyle. Interviewees see potential in using digital self-tracking to reassure anxious patients, that is, to confirm that there is nothing to worry about.

Still, a majority of our interviewees emphasize that every form of self-care is context-dependent in relation to the specific patient and his or her education and diagnosis. They are careful and nuanced when describing the advantages of the transformation toward a patient-manager and often point to concerns and pitfalls arising with this evolution. We will focus on some of these concerns. Some doctors worry that not every patient is sufficiently skilled to interpret medical data. Another concern focuses on how the “management” role of patients might lead to ignorant self-diagnosis, which already poses problems with classic forms of self-measurements.

There is the danger that patients will play doctor themselves. They will themselves decide whether or not to increase their blood pressure medication or diuretic pill.

1, GP

Doctor 12 (a cardiologist), however, believes this is not a major problem, as long as patients act within certain limits. For example, patients with diabetes already adjust their medication based on their daily self-tracking of blood sugar levels, which is described as a positive evolution.

Most doctors express concerns about how self-monitoring might lead to more distress and hypochondria by generating complex data and sometimes also information that the patient might not want to be confronted with. This concern reveals a tension with the aforementioned expected reassurance.

Another downside is the distress in patients, the problem is that he [the patient] cannot interpret the data himself, so he is alone at home and sees the results, but because of insufficient knowledge, he cannot assess the value of these [results].

3, GP

Another significant challenge is the high dropout rate. Our interviewees acknowledge that patients could be burdened with the self-tracking process and quit. Finally, five providers actively questioned the extent to which the apps and wearables actually *improve* health: there is a gap between measuring on the one hand and actual behavioral changes on the other.

I have the feeling that they do measure their parameters and that they are subsequently more aware of the problem, but [that] this does not really lead to behavioral changes.

4, GP

#### Theme 2: Health Obsession

When asked which type of patient takes the initiative to self-track, nine out of twelve informants describe how self-tracking is currently mostly initiated by patients who are already healthy: they worry or “obsess” about their health, or they use it to monitor sport activities. Even though GPs and cardiologists are not often confronted with patient-initiated forms of self-tracking, worried patients who do not actually need the tracking technologies for medical reasons most frequently ask questions. Most informants are concerned about this trend.

Although democratization—in terms of availability and easy accessibility of health apps and devices—is perceived as a good thing, most informants are not convinced that patients who are currently hard to reach will suddenly be reached with self-tracking technologies. Instead of fulfilling the promise of democratization, private digital self-care might establish a so-called *Matthew effect* [19]. In economics, this effect refers to the rich getting richer while simultaneously the poor become poorer. In a medical context, this means that the already healthy population might become even “healthier,” whereas the ones who would benefit most from self-monitoring are harder to reach. This raises compelling questions about health disparities.

There is an important Matthew effect: those who should not measure, measure, whereas those who do not measure, should measure. Consequently, a lot of money is going to those who do not actually need it, and those who do need it, are not getting it. That is the major problem. The overprotective and already well-controlled patients track themselves and the others do not.

7, GP

Yes, I expect that health disparities might increase because those who will use it [self-tracking tools] are the ones that are already part of the privileged class.

2, GP

This also intersects with the concern that the wider dispersal of digital self-tracking technologies might lead to increasing medicalization, that is, framing nonmedical issues, problems, or behaviors in terms of medical problems [20]. Informants are, for instance, hesitant about healthy people who monitor their recreational exercises such as weekly jogging because this monitoring might shift toward medicalization.

Currently, the data of “private” self-tracking are generally not shared with professional medicine, that is, our informants are only rarely confronted with patients who share these data with their care providers. The emerging “parallel circuit” of digital self-care in a home context raises questions about so-called “entertainment medicine” and an overabundance of medically unnecessary data that belong more to the fitness or wellness than to the medical realm.

On the one hand I know it [digital self-tracking] will be very useful for certain groups that we currently do not sufficiently reach. But then again, I notice that people who have these technologies now come here to whine...Well, whining might not be the right word...But with these apps you perform a whole lot of ‘entertainment’ medicine.

7, GP

Distinctions are subsequently made between self-tracking for fun or motivation versus medical necessity. Having easy access to a visual overview of one’s performances might work as a “motivational tool” (9, cardiologist), but these forms of self-monitoring are generally not medically necessary.

#### Theme 3: Information Management

This theme focuses on data: self-tracking methods change the way in which data are being generated and interpreted, and this impacts both the patient and the medical practice. In this context, providers discuss the devices and the data, the importance of context, and the extended possibilities for data sharing.

Most interviewees express their struggle with identifying high-quality devices, both in classic and digital material. With regard to classic self-tracking such as a blood pressure monitor, the informants often compare the devices of the patients with their own calibrated devices to look for deviations. Others use lists of approved brands provided by medical organizations. Yet, with regard to digital self-tracking, there are no lists available up to this point. Subsequently, there are high error margins and deviations in quality, reliability, and validity and an overall lack of evidence-based devices.

There is also the labeling of these devices. The government must absolutely develop a regulatory framework. This is extremely important, also in terms of technological development. I am very much looking forward to this framework. I notice that a lot of these devices are of poor quality.

7, GP

Certainly, a major problem concerns the validity of these data. That is the basis. With regard to the measurements done with Polars or iWatches, it remains uncertain to what extent these are correct. But these incorrect data are sometimes a reason that patients ask for a consultation. These patients are here because of an incredibly high measurement, but it is unsure whether there is a real problem or just an error.

10, cardiologist

Our interviewees are ambivalent about the data overload created by these devices. On the one hand, all informants are critical of this data overload if digital self-care would become an integrated part of professional health care. On the other hand, they acknowledge that more data can lead to more insight into medical conditions. In contrast to a single measurement that takes place at the doctor’s office, digital self-care can result in improved diagnosis, enhanced chronic care, and better preventive care.

Still, another challenge that has an important effect on data interpretation is the lack of context. One example is the fact that devices can potentially contain data of other people. Doctor 12 (a cardiologist) is involved in a project in which heart rhythm is measured with a mobile phone. He once gave the technology to an acquaintance of his who was interested in testing the technology but did not suffer from a heart condition.

At one point, a Saturday evening at 11 pm, I received an e-mail that contained a deviated heart rate measurement. I think ‘hmm, this is strange.’ So I send him [the acquaintance] an e-mail and he lets me know that he was at a reception, where he met someone who said that he suffered from a heart rhythm disorder and he [the acquaintance] subsequently gave him his smartphone to try the technology.

12, cardiologist

Being unaware of the specific context of the measurement might be potentially dangerous, especially if therapeutic decisions have to be made. This example shows that information always has to be interpreted within a given context.

A final subtheme of information management is data sharing. The diffusion, or sharing, of data, generated from digital self-tracking, can be divided into two categories. First, the self-tracker who shares data on social media, often to inform the network about their progress and to seek motivation. Informants express concerns about privacy infringement and receiving badly informed health advice from members of one’s online network. Second, data can be shared with the doctor and, in turn, with other health care providers through EHRs. If the right balance between useful and unnecessary data can be attained, more centralized data in EHRs could lead to better cooperation and communication with other care providers, a better chronic care, and enhanced quality of care. Problems with privacy, control, and user-friendliness should be tackled first because it requires technological complexity to interpret the data and upload them in the EHRs.

It is not just compatibility. It is about privacy. Who has the right to upload data in these files? And who has the right to delete them? It is also about all these questions. It is not that simple.

8, cardiologist

#### Theme 4: Shifts in the Roles of the Doctor and Impact on the Health Care Organization

Digital self-tracking might lead to changes in the roles of the physician in terms of becoming a coach at-a-distance and a data interpreter. Respondents are concerned about the “invasion” of digital self-care data into their practice, questioning the feasibility of the interpretation and usefulness (cf. “entertainment medicine”). Doctor 4 (a GP) draws an analogy between self-tracking and taking a blood test. Instead of only checking those parameters that are required to obtain an answer to a specific medical question, you would just check *all* the parameters the laboratory can possibly examine. As a result, you lose overview and context.

If you are going to fill in everything on this form, you will get such a complex picture, with so many results whose outcomes already complicate the interpretation of the question. I believe this situation is similar to self-measurements.

4, GP

Some informants express concern about taking responsibility for the data interpretation, given the threat of data overload and a constant flow of data. If patients share the collected data with their doctor, the interpretation of the data and appropriate response becomes the responsibility of the doctor.

If I receive all this [these data], I am responsible. It is the same with a blood test: if I have not looked at the data, it is my fault. While if I never received the data, I cannot be held responsible.

2, GP

Contrary to self-tracking technologies, no doctor is “always on” to interpret the data, and data interpretation can be time consuming. In *ideal* circumstances, all these data would improve the medical practice, making more time available for an in-depth conversation with the patient. Yet, instead of reducing costs and time investments, the risk exists that data from home monitoring may increase them.

The cardiologists in our sample already encounter problems with data overload in their daily practice. Doctor 8 (a cardiologist), who has experience with home monitoring and implanted defibrillators, observes that they lead to data overload: it is not just registration and monitoring, but the technologies also require data interpretation and responding to it, which is a job that takes 24/7. She states that more cardiologists are required at the hospital to deal with this extra work. Other informants also talk about the need of extra staff such as “telenurses” in a data control room. If paramedical teams can do the first analyses, physicians only become involved in case of actual problems.

When asked, none of the informants were concerned that their authority might be threatened. Most concerns relate to the flow of data and the decrease of “in-person contact” with patients in future medicine.

I did not study medicine to sit behind my computer. To have a conversation with the patient will regain importance.

2, GP

However, many providers acknowledge that the interaction with the patient will change in the sense that doctors will have to learn to deal with the patient as “patient-manager.” They, among others, point at the more proactive role of patients.

The physician must of course be able to deal with this. The generation of physicians, one or two generations ago, started from the model of the dominant doctor: the patient had to listen and the doctor was always right. We already see that this occurs less frequently. Patients ask more questions and discuss more with us, such as discussions related to the treatment.

11, cardiologist

This does not mean that our interviewees do not expect benefits from self-tracking for the health care organization. The professionalization and technical automation of self-care might significantly improve professional health care on the condition that problems and challenges are adequately addressed.

Especially with chronic patients: that if I make home visits to them, I already have an overview of their self-measurements from the past two weeks, or the last month before I leave. So I can check or look for the best next step, instead of waiting at their home until they have found their written notes or the notes that the nurse wrote down, often in a rush.

1, GP

They generally expect the benefits of digital self-tracking to be most obvious in preventive care. Prevention often requires lifestyle changes, and both doctor and apps could help the patient in reaching their individual goals. One cardiologist acknowledges that there is a lot of work to be done on the level of prevention.

Regarding acute treatments, not much improvement is required, but in the domain of primary and secondary prevention we can still improve a lot. Secondary prevention also includes lifestyle adjustments, and only the patient himself can attain this. So I think that patient engagement will become very important. And that the doctor becomes more of a coach, instead of a dominant person. And actually, that is the legitimate role of the physician.

11, cardiologist

## Discussion

### Bridging Themes and Literature

The conducted interviews paint an image of new opportunities and challenges instigated by the wider dispersal, accessibility, and affordability of self-tracking tools. Our research findings reveal that self-tracking is expected to lead to shifting understandings of professional care and of the patient–care provider relationship, which is in accordance with other studies’ findings. In what follows, our principal findings are discussed in relation to these studies.

### The Healthy, Empowered Patient?

Providers express the expectation that increased self-tracking will lead to more patient empowerment: in their view, self-management of health will gain importance. At the same time, they raise critical questions about the actual results and the possible harms this role can cause to the patient. These two critical points deserve further scrutiny.

#### From Self-Tracking to Behavioral Change

Five providers describe awareness of a gap between measuring on the one hand and actual behavioral changes on the other. In this context, two questions are currently discussed in the existing literature but remain largely unanswered. First, can wearables affect healthy users’ behavior and promote lifestyle changes? The scientific evidence for this effect is meager [7]. Self-tracking devices are most often used as consumer gadgets, and it remains unclear to what extent they can benefit public health. A recent randomized controlled trial examined the effect of adding wearable technology to a weight loss program [21]. The behavioral intervention turned out to become less effective for 24-month weight loss with the addition of wearable technology. Although there were some limitations to the technology used in this study (eg, the device was worn on the upper arm instead of on the wrist), other studies also struggle with proving long-term effects and face a high dropout ratio. Another recent study examined the feasibility of obtaining measures of cardiovascular health—such as physical activity, fitness, and sleep—via a mobile phone app. Although the observation period in this large-scale study was limited to 7 days, only 9.30% (4552/48,968) of participants who consented actually completed that period [22]. These studies confirm the doubts that our providers express of how to bridge the gap between measuring and an actual sustained behavioral change. Moreover, self-tracking takes place at the individual level, whereas most lifestyle-related problems come with a strong societal connection. Obesity, for example, keeps on increasing for many reasons including an abundance of cheap and unhealthy food and widespread marketing campaigns of the food industry [23]. Deep-seated problems such as obesity cannot be easily fixed by means of self-tracking because they require a structural societal approach.

A second question is whether the use of consumer health apps and devices can improve the outcomes of patients’ self-management of chronic diseases. Up to this point, the many expectations about the quantified self for health care are not yet fulfilled [24]. A review study focusing on self-management interventions of rheumatic diseases describes the potential of mobile phone apps but also several pitfalls: scientific evidence for the apps was often lacking, their use can be limited by education level, and the continuous utilization can cause many problems. It concludes that—although online stores offer several apps—more scientific research is needed on the development of such apps [25]. Another meta-analysis focusing on diabetes “identified significant, yet small, reductions in the HbA1c, blood pressure, total cholesterol, and triglycerides levels of patients who were involved in the technology-integrated disease self-management groups” [26]. However, some other patient outcomes did not improve, and it remained unclear why effectiveness was not observed [26]. Although patient empowerment is often read as a precursor to better care and better health, both our interview study and the existing literature point toward a more nuanced view and poses questions about the sheer certainty with which this is accepted.

#### Patient Harm

This leads to the second part of our discussion of the interviewees’ critique: could the role of health-manager cause damage to the patient? Again, we want to focus separately on the healthy individual and the chronic patient. The majority of our interviewees express concern about a focused use of self-tracking by healthy people, creating a “worried well” cohort and widening health disparities. Our finding that mainly already healthy individuals are interested in self-tracking is in accordance with other findings, which shows that mostly young people use smart wearable devices [27]. However, some of the providers see an opportunity here: a small group of early adopters could pave the way toward broader access in the population. The literature, as well as our providers’ impression, confirms that digital disparities exist in the adoption and utilization of digital self-care [28]. The aforementioned study focusing on cardiovascular health provides an interesting example, as young male individuals were heavily overrepresented in their study population: of the almost 49,000 individuals consenting to participate, 82.2% were male with a median age of 36 years [28]. Although health IT applications have the potential to address existing health disparities, this requires surmounting several significant technical, practical, and human challenges [28].

With regard to the aforementioned shift toward the patient-manager, informants are critical of the broader societal trend to manage and medicalize health. This excessive emphasis on health is rated undesirable [29,30]. In the literature, it has been described as the “medicalization of health and life itself” [30]. Health is no longer labeled as the absence of disease but has itself become medicalized. This particular understanding of health is linked to the emerging concept of personalized medicine. Many of the digital self-tracking devices are developed within the logic of personalized medicine. The continuous monitoring of healthy people can lead to data overload, overdiagnosis, and overtreatment. The labeling of various aspects of life as “medical” may lead to displacing other values, and the state of being healthy may become impossible to reach [30].

Our providers recognize the increased importance of self-management of chronic diseases while acknowledging the risks that accompany the role of health-manager for patients. For example, nine interviewees explicitly raise concern about increasing distress in patients through self-tracking. They wonder whether patients possess the necessary skill set to deal with the data. Another interview study focusing on patients with multiple chronic conditions raised similar concerns [31]. Two of their findings are of particular interest to our study. First, personal data tracking could carry strong emotional and moral implications: data provoked personal judgments (“good” or “bad”) and sometimes negative feelings (“depressed” and “scared”). Second, many patients described self-tracking as a time-consuming effort, even work. In this context, the problem of dropout was also addressed in our study. This concern led some of our interviewees to turn to more paternalistic solutions: to prevent harm, they proposed to limit the patient’s autonomy in self-tracking and therefore, to limit the burden of decision making. We question whether this reflex will provide the right answer to the presented challenges; perhaps the solution would be to move forward toward a reviewed concept of patient autonomy. This, however, is beyond the scope of this paper.

### Impact on Patient-Provider Relationship

Studies on digital self-care, conducted in the context of experimental studies in Flanders (Veys et al, IBBT TranseCare: deliverable 4.6, 2009, unpublished material) [32], show that patients still value the doctor’s expertise but seek more participation. Patients involved in the studies strived for a balanced and reciprocal relationship. They wanted to have a consultation and dialogue with an expert who interprets the data. Although self-tracking could lead to more patient participation, a risk that should be anticipated is that increased patient participation could result in shifting the burden of responsibilities and agency onto patients [33,34]. The focus on self-management, facilitated by digital technology, could damage the clinical relationship by emphasizing the importance of biomedical outcomes in clinical interactions [34].

### Overarching Concepts

Finally, we want to discuss two important notions that arose in all our interviews. Although sometimes retreated in the background of the providers’ narratives, these notions form an essential part of the nuances that came to the fore in our study. The concepts of context-dependency of health care and the professionalization of self-care are essential to develop a thorough understanding of the providers’ report.

#### No One-Size-Fits-All

The promises and expectations of digital self-care are often grounded in a model of ideal situations. Among other things, they focus on patients who have the right knowledge and adequate skills to interpret the data and to deal with the challenges of these new technologies and who also have financial resources and social opportunity to act on the information. A shared concern of our informants is that this alleged democratization of self-care neglects the context-dependency and complexity of everyday medical practices. Specific uses and success rates are always dependent on multiple variables and patient conditions. Additionally, our research findings show that our informants would not advise *every* patient to track. For example, patients who are expected to suffer from more distress or hypochondria when confronted with all these data would be discouraged to self-track. To facilitate the positive development of digital self-care, academic literature should move away from focusing on “ideal” situations and reflect on more context-relativity and complexity. Both patients and health care providers need guidance regarding these shifts that the digitalization of self-tracking and self-care bring about.

#### Professionalization of Self-Care

Today, patients are provided with more technologically advanced tools to manage their health. In line with other studies [35], our study illustrates that the boundary between self-care and professional care is decreasing. Kielmann et al [35] focus on the viewpoint of patients, a perspective that our study’s focus complements, and show that patients can feel abandoned by the care providers. The patient-care provider relationship must always be a two-way process in which the active role of the patient is acknowledged. Our findings reveal that our informants are critical of not only the quality of these apps and devices but also the reliability of the measurements that the patients themselves have to do.

Physicians should not retreat from this evolution toward patient self-tracking but take extra training to deal with data interpretation and to inform their patients. Not all patients are aware of the added value that these technological developments can offer them (eg, sleep monitoring app vs sleeping pills). A good practice of digital self-care requires patient education and digital literacy.

### Limitations of This Study

A number of shortcomings of this research must be acknowledged. First, this study addressed a broad theme by incorporating both classic and digital forms of self-care to make their comparison possible. Second, our small sample, although necessary to develop a rich, detailed description, limits our ability to make wider claims based on these interviews. This study explores relatively new phenomena in-depth; further research is necessary to consolidate our findings (cf. infra). Third, we only studied physicians and did not incorporate the viewpoints of patients. Overall, in our small-scale study, we were particularly attentive to implement a number of best practices such as the use of multiple coders, attention to deviant cases, member validation, and a constant comparative technique (cf. supra).

### Concluding Remarks and Future Research Endeavors

This study sought to investigate the shifting understandings in medical practice that “private” self-tracking is expected to engender. In so doing, we studied the potential and challenges of self-monitoring while focusing on the everyday professional experience of the physician. Our study offered an analysis of how a small sample of Belgian GPs and cardiologists evaluate self-tracking methods to explore the anticipated shifts. Overall, our research findings showed a nuanced understanding that is in accordance with existing literature on digital self-care.

Our findings open interesting opportunities for other studies. First, our research results can be used for further conceptual scrutiny, especially concerning the presented theme of the patient-manager and the meaning of autonomy in professional health care. As our informants emphasized, the burden of responsibility could prove harmful to some patients. A second interesting path for future research is to explore patients’ experiences with digital self-tracking. A main question stemming from our study is whether and how they integrate the data in the patient-doctor relationship. Such studies need to be attentive to context and actual lived experiences. Third, it would be interesting to translate our discussed themes and subthemes to a quantitative questionnaire to reach more doctors and to examine to what extent they share the same ideas. Finally, after analyzing empirical data from both provider and patient perspectives, ethical frameworks should be developed to adequately address the challenges of medicalization of health and professionalization of self-care.

## Appendix

Multimedia Appendix 1 Informed Consent form.

Multimedia Appendix 2 Interview guide.

Multimedia Appendix 3 Coding Scheme.

## Abbreviations

EHR: electronic health record
GP: general practitioner
mHealth: mobile health
