# Supplementary material for: Exploring Entertainment Medicine and Professionalization of Self-Care: Interview Study Among Doctors on the Potential Effects of Digital Self-Tracking
Source: J Med Internet Res. 2018 Jan 12;20(1):e10. doi: 10.2196/jmir.8040 (PMC5786746; doi:10.2196/jmir.8040)
Supplement: Multimedia Appendix 1 [file jmir_v20i1e10_app1.pdf]

APPENDIX 1: Informed Consent Form

Thank you for your willingness to participate in this interview study concerning self-tracking in the general practice. You already received information on this study in the background letter we provided. We will record this interview for future analysis. After completing this analysis, the recordings will be destroyed. Names of participants will be anonymized, and all personal identifiers will be adjusted or deleted. Only the principal investigators of this study have access to the raw data.

The results of this study will be presented at conferences or workshops and published in scientific journals. You will never be identified in these presentations or papers. In case you would like to receive the results, you can send us an e-mail.

In case you have any further questions or comments, do not hesitate to discuss these beforehand.

Please sign this form by ticking every box in case you agree with the following:

1) I confirm that I have read the information above, and that I was offered the opportunity to ask questions.
2) After reading the information, I declare that I voluntarily participate in this research project. I understand that I can withdraw from the study at any time, without explaining the reason.
3) I allow this interview to be recorded.

Name:
Date:
Autograph:
