# Supplementary material for: Exploring Entertainment Medicine and Professionalization of Self-Care: Interview Study Among Doctors on the Potential Effects of Digital Self-Tracking
Source: J Med Internet Res. 2018 Jan 12;20(1):e10. doi: 10.2196/jmir.8040 (PMC5786746; doi:10.2196/jmir.8040)
Supplement: Multimedia Appendix 2 [file jmir_v20i1e10_app2.pdf]

APPENDIX 2: Interview Guide

A short, paper-based questionnaire was completed after signing the informed consent. We first asked for general information:

- age
- gender
- type of practice
- years in practice
- city of residence
- IT use in practice

We provided background information, explaining classic and digital self-tracking with examples. We then attempted to gauge understanding:

- What does e-health entail according to you?
- …Telemonitoring or telemedicine?
- Do you personally use self-tracking apps or devices?
- Do you use any for your patients?
- Which digital self-tracking apps do you know about? How did you get to know about them?

We inquired about the current situation in the practice: "I will now ask you some questions to understand how you use and appreciate the classic self-tracking devices. Good examples to keep in mind are blood pressure meters, oxygen sensors, and glucose meters."

- *frequency*:
  - How frequently do you use this type of self-tracking in your practice? Estimate per week.
  - How often are you confronted with questions about this type of self-tracking?
- *quality*:
  - What kind of quality measures should these devices attain?
  - How do you distinguish between good and bad quality?
- *initiative*:
  - Do patients take the initiative to track? What type of patients do you think track most often?
  - In what kind of situations do you advice patients to self-track? What type of patient would you encourage or discourage to track?
- *data interpretation:*
  - How do you cope with interpreting data from these devices?
  - Do you feel like you have enough knowledge/understanding to do this?
  - Would you enroll in continuing education for this?
  - How do you organize the data interpretation in your consultation schedule?
  - How much time does this require?
- *effects/results*:

- Do you think self-tracking is a positive evolution? In which sense could data from self-tracking lead to better care?
- What problems or pitfalls can you identify?

We moved into talking about digital self-tracking. “New apps, usually smartphone-based, are being developed at a fast pace. In the second part of this interview, we would like to talk about your experience in the general practice with this type of self-tracking, and explore your views.”

- *frequency*:
  - How frequently do you encounter this type of self-tracking in your practice? Estimate per week.
  - How often are you confronted with questions about this type of self-tracking?
- *quality*:
  - What kind of quality measures should these devices attain?
  - How do you distinguish between good and bad quality?
- *differences from classic and self-tracking*:
  - What differences can you discern?
  - Do you observe digital self-tracking in your daily clinical practice?
- *initiative*:
  - One of the differences that has been described is that healthy people are more often using digital self-tracking apps. Is this something you encounter?
  - In what situation would you recommend digital self-tracking to patients?
- *impact*:
  - What kind of impact do you expect digital self-tracking to have:
    - ...On your general practice?
    - ...On your role as general practitioner/cardiologist?
    - ...On the patient-doctor relationship?
  - Do you expect this technology to increase or decrease trust in medicine? (*prompt*) If you expect change, why? (*specify*)
- *data sharing*:
  - Patients can share self-tracking data online: for example, through social media. Do you think this comes with mostly opportunities or problems? (*prompt: e-patient Dave*)
  - Data can be shared electronically with the doctor: for example, uploaded into the EMR. How do you feel about this? What effects would you expect, positive or negative? How could this impact your consultations?
- *inclusion*:
  - Do you expect that most patients will come to use digital self-tracking over time?
  - Do you expect an impact on health disparities?
- *coaching:*

- Apps are being used increasingly to coach the user, with the goal of attaining behavioral change (*refer back to examples*). Do you think the apps could be added value to your practice?
- Do they pose a threat?

We closed by asking about the future. “Imagine your practice in 2026: ten years from now.”

- What evolution do you expect in the level of digital self-tracking apps?
- What will medicine look like ten years from now?
- How do you feel about this evolution?
