# Supplementary material for: Exploring Entertainment Medicine and Professionalization of Self-Care: Interview Study Among Doctors on the Potential Effects of Digital Self-Tracking
Source: J Med Internet Res. 2018 Jan 12;20(1):e10. doi: 10.2196/jmir.8040 (PMC5786746; doi:10.2196/jmir.8040)
Supplement: Multimedia Appendix 3 [file jmir_v20i1e10_app3.pdf]

APPENDIX 3: Coding Scheme

patient as manager	opportunities	motivation	
		behavioral change	
		therapy adjustment	
		self-care	
		reassurance	
		increased insight/understanding	
		screening	
	concerns	distress	
		unsustained motivation	
		only a “happy few” benefit	
		not for the elderly	
		increased health gap	
		false reassurance	
		confrontation	
		drop-out	
		interpretation of data	
		loss of autonomy	
		therapy adjustment	
	factors	computer literacy	
		patient variability	
		need for guidance and education	
health obsession	who takes the initiative	worried well	
		sports and exercise enthusiasts	
	concerns	“Matthew effect”	
		device dependence	
		time investment	
		“Quantified Self”	
		medicalization	
		unnecessary examination	
		failure to change behavior	
	opportunities	behavioral change	
		screening	
		patient safety	
		better quality of health	
	commercial health apps	“entertainment” medicine	
		lack of evidence-based medicine (EBM)	
		overabundance of apps	
information	devices	concerns	complexity

management			data quality
			lack of norms and regulations
			privacy, security
		coping strategies	comparison across devices
			manufacturer, brand
			professional recommendation
		factors	use of EBM
			accuracy
			government labeling
	data	concerns	quality, accuracy
			variation across devices
			patient interpretation
			information overload
			lack of context
			complexity
			impact of automation
		opportunities	more data
			better quality data
			telemonitoring
			revalidation
			automation potential
			patient safety
			better diagnostics and screening
		factors	patients' level of education
			information relevance
			use of EBM
			algorithms employed
			information context
			legal framework
	data sharing	social media	support group
			patient motivation
			risk of bad advice
		electronic health record (EHR)	more data
			decision support
			chronic care
			source identification
			more relevant data

			time consumption
			risk of data overload
care provider's shifting role	doctor	concerns	time investment
			fear of being "always on"
			responsibility for data
			data overload
		opportunities	time savings
			automation
			opportunity for being "always on"
		factors	feedback to doctor
			patient communication
			doctors' education
			financial compensation
	expected impact on professional care		
	impact on patient-doctor relationship	trust	
		personal relations	
		risk for paternalism	
	expected impact on physician authority		
	frequency of consults	increase?	
		decrease?	
	financial impact	burden?	
		saving?	
	self-tracking as supplement, not replacement		
